# Single prolonged stress induces behavior and transcriptomic changes in the medial prefrontal cortex to increase susceptibility to anxiety-like behavior in rats

**DOI:** 10.3389/fpsyt.2024.1472194

**Published:** 2024-11-19

**Authors:** Javed Iqbal, Geng-Di Huang, Dan Shen, Yan-Xue Xue, Mei Yang, Xiao-Jian Jia

**Affiliations:** ^1^ Department of Addiction Medicine, Shenzhen Clinical Research Center for Mental Disorders, Shenzhen Kangning Hospital and Shenzhen Mental Health Center, Clinical College of Mental Health, Shenzhen University Health Science Center, Affiliated Mental Health Center, Southern University of Science and Technology, Shenzhen, China; ^2^ Department of Neurobiology and Behavior, Stony Brook University, Stony Brook, NY, United States; ^3^ Henan Key Laboratory of Medical Tissue Regeneration, Xinxiang Medical University, Xinxiang, Henan, China; ^4^ National Institute on Drug Dependence and Beijing Key Laboratory of Drug Dependence, Peking University, Beijing, China

**Keywords:** traumatic stress, prefrontal cortex, susceptibility, resilient, anxiety

## Abstract

**Introduction:**

Transcriptomic studies offer valuable insights into the pathophysiology of traumatic stress-induced neuropsychiatric disorders, including generalized anxiety disorder and post-traumatic stress disorder (PTSD). The medial prefrontal cortex (mPFC) has been implicated in emotion, cognitive function, and psychiatric disorders. Alterations in the function of mPFC have been observed in PTSD patients. However, the specific transcriptomic mechanisms governed by genes within the mPFC under traumatic stress remain elusive.

**Methods:**

In this study, we conducted transcriptome-wide RNA-seq analysis in the prelimbic (PL) and infralimbic (IL) cortices. We employed the single prolonged stress (SPS) animal model to simulate anxiety-like behavior, which was assessed using the open field and elevated plus maze tests.

**Results:**

We identified sixty-two differentially expressed genes (DEGs) (FDR adjusted *p* < 0.05) with significant expression changes in the PL and IL mPFC. In the PL cortex, DEGs in the susceptible group exhibited reduced enrichment for cellular, biological, and molecular functions such as postsynaptic density proteins, glutamatergic synapses, synapse formation, transmembrane transport proteins, and actin cytoskeleton reorganization. In contrast, the IL-susceptible group displayed diminished enrichment for synapse formation, neuronal activity, dendrite development, axon regeneration, learning processes, and glucocorticoid receptor binding compared to control and insusceptible groups. DEGs in the PL-susceptible group were enriched for Kyoto Encyclopedia of Genes and Genomes (KEGG) pathways related to Parkinson’s disease, Huntington's disease, Alzheimer's disease, and neurodegeneration processes. In the IL cortex, the susceptible group demonstrated enrichment for KEGG pathways involved in regulating stress signaling pathways and addiction-like behaviors, compared to control and insusceptible groups.

**Conclusion:**

Our findings suggest that SPS activates distinct transcriptional and molecular pathways in PL and IL cortices of mPFC, enabling differential coping mechanisms in response to the effects of traumatic stress. The enhanced enrichment of identified KEGG pathways in the PL and IL mPFC may underlie the anxiety-like behavior observed in susceptible rats.

## 1 Introduction

Post-traumatic stress disorder (PTSD) is a complex and chronic mental disorder associated with stress, characterized by an imbalance of neurotransmitters in response to traumatic events or fears (1). PTSD presents as a heterogeneous array of symptoms in response to traumatic life events, including anxiety, re-experiencing, irritability, avoidance, negative emotions, insomnia, personality changes, and memory problems (2). A PTSD diagnosis is given when these symptoms persist for at least one month, causing functional impairment and distress. The National Health Center of PTSD in the United States estimates that 3.5% of the US population (over 11 million Americans) experience PTSD each year, but less than half of these individuals receive proper medical treatment, and even fewer receive minimally adequate care (3). PTSD-related neural disruptions encompass asymmetrical white matter tract abnormalities and gray matter alterations in the prefrontal cortex (PFC), hippocampus, and basolateral amygdala (BLA) (4). Dysfunction within this neural circuitry results in behavioral changes, including executive function and memory impairments, fear retention, fear extinction deficiencies, and other disturbances.

Various animal models have been employed to replicate the symptoms of PTSD (5). The single prolonged stress (SPS) model, a suitable animal model for PTSD, has been developed to investigate the neurobiological mechanisms underlying PTSD (6). Prior studies have utilized animal models to characterize a fear learning and memory retention network centered on the PFC, hippocampus, and amygdala, which are crucial in the pathology of PTSD (7, 8). Notably, structural, functional, and biochemical changes in these brain regions causes dysfunction of cognitive abilities observed in PTSD (9).

The PFC plays an important role in decision-making and executive functions, including attention, working memory, and regulation of emotional behaviors (10, 11). The function of three PFC subregions, the anterior cingulate cortex (ACC), prelimbic cortex (PL), and infralimbic cortex (IL), is altered in PTSD (12, 13). The PL and IL regions specifically contribute to fear conditioning and extinction processes. The human ventromedial PFC (vmPFC) plays an essential role in the extinction of fearful memories by interacting with the amygdala to inhibit fear expression (14, 15). The disruption of vmPFC function impaired the retention of fear extinction learning in PTSD individuals (16, 17). Decreased vmPFC activity has been observed in PTSD individuals experiencing traumatic symptoms (18). Previous transcriptomic studies revealed expression changes in glucocorticoid and neuronal pathways involved in regulating the PTSD-related behavioral responses (19, 20). A recent study has identified transcriptomic changes within the locus coeruleus (LC) and nucleus accumbens (NAc) associated with stress susceptibility or resilience behaviors (21). Another study showed extensive remodeling of transcriptome occurred in the PFC of PTSD individuals and identified genes involved in GABAergic signaling (22). None of these studies showed any differential role of the transcriptome of subregions of the PFC in stress susceptibility or resilience phenotypes. We investigated this question at the transcriptional level by examining the transcriptome of PL and IL regions and providing an in-depth analysis of the regulated pathways that contribute to the distinct roles of the PL and IL in traumatic stress-induced anxiety-like behavior. We hypothesized that single prolonged stress alters animal behavior and induces anxiety-like behavior by differentially regulating gene expression in the mPFC. The primary objective of this study was to assess whether transcriptional changes in the PL and IL regions elicit differential responses in the regulation of anxiety-like behavior in stressed male rats. Although numerous studies have documented sex differences in stress responses, particularly in behavior and gene expression, in both rodents and humans (23–26). We have used only male rats in this study due to the fact that the fluctuations in estrogen hormones might impact the behavioral and the transcriptomic profiles in stress condition (27–30).

## 2 Materials and methods

### 2.1 Experimental animals

For all experiments, twelve-week-old male Sprague Dawley rats were utilized. The rats were housed in a temperature-controlled (22 ± 2°C) and humidity-controlled (50–60%) animal care facility within the institution, maintained on a 12:12-hour light and dark cycle (lights on at 7:00 AM), and provided unrestricted access to ad libitum food and tap water. The institutional ethical committee approved the animal protocol for this study (Protocol number: SL2022022413). All experiments were conducted in accordance with the ethical principles of animal use and care. Following a seven-day acclimatization period, the animals were randomly assigned to either the control (n=10) or stress (n=20) groups.

### 2.2 Single prolonged stress (SPS protocol)

The SPS procedure was performed over ten days, as previously described in our study (31). Animals in the stress group received SPS for ten days and were subsequently categorized into susceptible (sus, n=11) and insusceptible (insus, n=9) subgroups based on their phenotypes and anxiety index (AI) in behavioral experiments. Briefly, all stressed animals were sequential exposed to three stressors (2 h of restraint, a 20-min forced swim, and exposure to ether until loss of consciousness) during a single continuous session. The three stressors were always conducted in the same order and at the same time of day. First, all stressed animals were immobilized for two hours inside a restrainer that restricted body movement. Subsequently, they were subjected to a 20-minute forced swim in a plexiglass cylinder (50 cm high, 24 cm diameter) filled to two-thirds with 24°C water. The animals were dried and allowed a 15-minute recuperation period before being exposed to ether vapor until the loss of consciousness. On the same day of SPS treatment, the control animals were handled. The following behavioral tests were performed 24h after the last SPS day.

### 2.3 Elevated plus maze test

Each rat was placed in the central square (10 × 10 cm) facing two open arms (50 × 10 cm) and two closed arms (50 × 10 × 40 cm) in the EPM apparatus. Rats were allowed to freely explore the four arms for five minutes (32). A valid entry into any of the four arms was recorded via video-computerized tracking system when all four paws of a rat crossed from the central region into an arm and analyzed with LabMaze v3.0 (Zhongshi Technology, China). The total number of open arm entries and the duration spent in the open arms by each rat were recorded.

### 2.4 Open field test

Before the OFT, all rats were placed in the animal test room for an hour. Each rat was then positioned at the center of the open field box (60 × 60 × 60 cm chamber) and allowed to explore the arena freely for a 5-minute test session (33). A SMART video-computerized tracking system (SMART 3.0) recorded the locomotive activities of each rat during the OFT. The central portion of the testing area located in the middle of the larger arena was assigned as “center” of the arena in the OFT. The total distance and time spent in the center of the open field box were calculated for the 5-minute session. After performing the behavioral tests, stressed animals were retrospectively classified as having behavioral responses according to the anxiety index (AI), which is calculated as 1 - [(time spent in open arm/total time on the maze)/2 + (number of entries to the open arms/total exploration on the maze)/2]. For classification criteria, one standard deviation below the mean of the control group anxiety index as resilient individuals, and one standard deviation above the mean of the control group anxiety index as susceptible individuals. After SPS and behavioral tests, 11 rats reached susceptible criteria and 9 rats reached resilient criteria. For RNA-Seq, four animals from each group were selected based on the AI in their respective group. Carbon dioxide (CO2) inhalation method followed by cervical dislocation was used for harvesting the brains. All animals were sacrificed 24h after the OFT and EPM tests, and brain samples were dissected from each animal. Then, the mPFC tissue samples were sent to BGI Co., LTD (Shenzhen, China), and the BGISEQ-500 platform was used to perform RNA sequencing.

### 2.5 Extraction of mRNA and cDNA library preparations

The TRIzol RNA extraction kit (Life Technologies, Darmstadt, Germany) was used to extract all RNA samples followed by DNAse I treatment to eliminate any DNA contamination from the extracted samples (RNA Clean & Concentrator, Zymo Research, Irvine, CA, USA). After total RNA extraction, the Ribo-Zero™ Magnetic Kit (Epicenter) was used to remove rRNA from the total mRNA samples. The purity and concentration of mRNA were determined from the A260-nm/280-nm reading using a Nanodrop spectrophotometer. All samples used for RNA sequencing experiments displayed RNA integrity numbers above eight. The extracted mRNA was reverse transcribed into cDNA using random octamer primers tagged with a 20-nucleotide tag sequence (5`-GACCATCGNNNNNNNN-3`). cDNA synthesis was carried out by adding DNA polymerase I, dNTP, RNase H, and buffer. Subsequently, the QiaQuick PCR extraction kit was used to purify the cDNA fragments, end-repaired, poly (A) tails added, and ligated to Illumina high sequencing adapters. The ligation products underwent size selection through agarose gel electrophoresis, followed by PCR amplification. Finally, the samples were sequenced using the Illumina HiSeqTM platform.

### 2.6 RNA sequencing data analysis

All raw reads underwent quality filtering and adapter trimming using the Trimmomatic (34) and FastQC (35) packages. All clean reads were mapped to the reference sequence using Bowtie2 (36), and the expression levels of genes and transcripts were calculated with RSEM (37). Gene expression levels were normalized using the FPKM (fragments per kilobase of transcript per million mapped reads) method. The DEseq2 method (38) was employed to identify differentially expressed genes (DEGs). Genes with a fold change ≥ 1.5 and a false discovery rate (FDR) < 0.05 were classified as DEGs. Statistical analysis was conducted using the R package gmodels. The identified DEGs were subsequently analyzed based on enrichment analysis.

### 2.7 Analytical method

Enrichment analysis was performed to determine whether a specific gene set was significantly enriched in a pathway, molecular function, or participating biological process. The gene sets of interest were DEGs or target genes differentially expressed between groups. DEGs were further examined through enrichment analysis, as detailed below.

#### 2.7.1 Gene ontology enrichment analysis

GO functional enrichment analysis identified GO terms significantly enriched in candidate genes compared to the entire genetic background of the species, thus revealing the biological functions significantly associated with the candidate genes. All candidate genes were mapped to the Gene Ontology database (http://www.geneontology.org/), and calculated the number of genes per entry. A hypergeometric test was used to identify significant GO functions enriched in candidate genes relative to all background genes of the species. The *p*-value was calculated for each GO functions using the hypergeometric test of R (https://stat.ethz.ch/R-manual/R-devel/library/stats/html/Hypergeometric.html). Subsequently, the *p*-value was corrected for multiple testing, and the corrected *p*-value, denoted as the *q*-value, was determined using the Bioconductor package (https://bioconductor.org/packages/release/bioc/html/qvalue.html). A *q*-value (FDR adjusted *p*-value) ≤ 0.05 served as the threshold, and GO terms meeting this criterion were considered significantly enriched in candidate genes.

#### 2.7.2 Kyoto encyclopedia of genes and genomes pathway enrichment analysis of identified DEGs

KEGG pathway-based enrichment analysis (39) facilitated a deeper understanding of the biological functions of DEGs. The KEGG analysis employed the same methodology as the GO functional enrichment analysis detailed above. The calculated *p*-values for GO terms and KEGG pathways underwent FDR correction, using FDR ≤ 0.05 as the threshold. Pathways with a final *q*-value < 0.05 were deemed significantly enriched in DEGs. These significant enrichment pathways highlighted candidate DEGs as crucial components in biochemical, metabolic, and signaling pathways. RNA sequencing was performed by BGI (https://biosys.bgi.com).

### 2.8 Statistical analysis

All data were normally distributed, and statistical differences among groups were assessed using one-way ANOVA followed by Tukey’s multiple comparison test. Combined data were presented as mean ± SEM. All statistical analyses were conducted using GraphPad Prism software (ver. 9.0; GraphPad Software Inc., San Diego, CA, United States). A statistical difference was considered significant at *p* < 0.05. For RNA-seq data, statistical analyses were performed using Cuffdiff2, edgeR, and DESeq2 methods.

## 3 Results

### 3.1 Traumatic stress produced anxiety-like behavior in males

**Figure 1:**
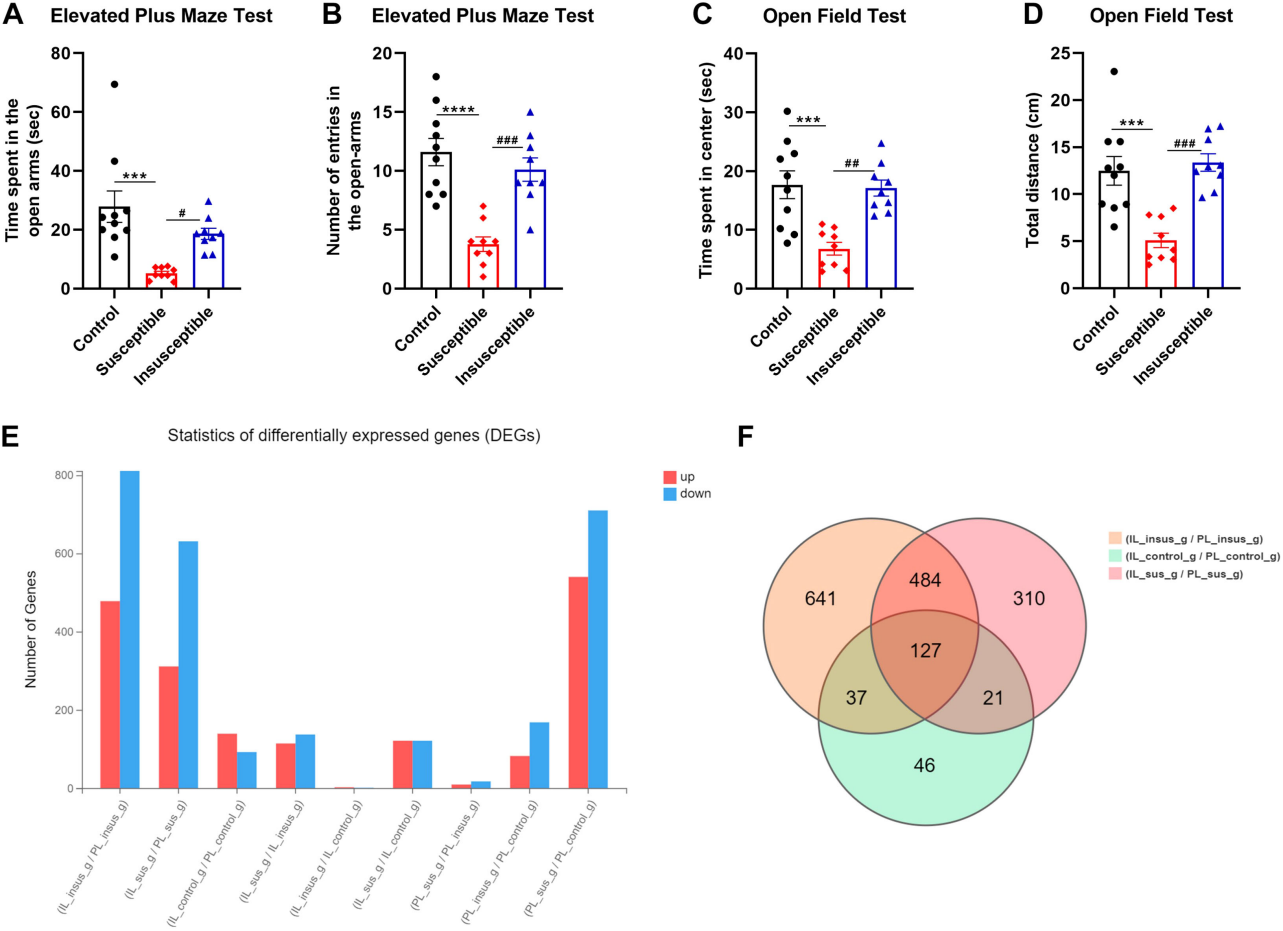
Anxiety-like behavior and transcriptional differences in the prelimbic (PL) and infralimbic (IL) cortex induced by single prolonged stress (SPS). **(A)** Open field test (OFT) results show significantly decreased exploratory activity in susceptible animals compared to control *(p = 0.002)* and insusceptible (*p* = 0.005) groups. No significant difference was observed between control and insusceptible groups (*p* = 0.89). **(B)** Susceptible animals exhibited significantly less time spent in the open field center compared to control and insusceptible groups (Control *vs*. Susceptible, *p* = 0.004; Susceptible *vs*. Insusceptible, *p* = 0.009). **(C, D)** In the elevated plus maze test, susceptible rats spent significantly less time in open arms (Control *vs*. Susceptible, *p* = 0.003) and had fewer open arm entries (Control *vs*. Susceptible, *p* = 0.0005). No significant difference was found between control and insusceptible groups in these measures (Time in open arms, *p* = 0.47; Open arm entries, *p* = 0.84). **(E)** SPS-induced transcriptional changes were examined in susceptible and insusceptible animals’ PL and IL regions of the medial prefrontal cortex. More transcriptional changes were observed in PL compared to IL, with the majority of identified differentially expressed genes (DEGs) being downregulated. **(F)** Venn diagram displays the total DEGs in each group and the overlapping DEGs among control, susceptible, and insusceptible groups. Control_g represents the control group; sus_g, susceptible group; insus_g, insusceptible group. Data are shown as mean ± SEM (*n* = 10 per group). ***p < 0.01; ****p < 0.0001; #*p* < 0.05; ##*p* < 0.01; ###*p* < 0.001.

We investigated the impact of SPS on animal behavior by conducting EPM and OFT to assess anxiety-like behavior induced by traumatic stress. SPS significantly reduced the time spent in the open arms (**Figure 1A**, F_2, 25_ = 10.74, *p* = 0.0004) and the number of open arm entries (**Figure 1B**, F_2, 25_ = 18.10, *p* < 0.0001) across control, susceptible, and insusceptible groups. *Post-hoc* analysis revealed that susceptible rats spent notably less time in the open arms compared to the control (**Figure 1A**, *p* = 0.0003) and insusceptible groups (**Figure 1A**, *p* = 0.0339). Likewise, susceptible stressed rats exhibited a significant difference in the number of open arm entries relative to the control (**Figure 1B**, *p* < 0.0001) and insusceptible groups (**Figure 1A**, *p* = 0.0004). No significant differences were observed in the time spent in the open arms (**Figure 1A**, *p* = 0.4793) and the number of open arm entries (**Figure 1B**, *p* = 0.5288) between the control and insusceptible groups.

The OFT evaluated the subjects’ innate exploratory behavior in an open field. SPS significantly influenced the time spent in the center of the open field (**Figure 1C**, F_2, 25_ = 11.89, *p* = 0.0002) and the distance traveled (**Figure 1D**, F_2, 25_ = 15.09, *p* < 0.0001) among control, susceptible, and insusceptible groups. *Post-hoc* analysis demonstrated that SPS substantially decreased the time spent in the center of the open field for susceptible animals compared to controls (**Figure 1C**, *p* = 0.0005) and insusceptible animals (**Figure 1C**, *p* = 0.0012). Susceptible animals covered significantly less distance in the open field than controls (**Figure 1D**, *p* = 0.0003) and insusceptible animals (**Figure 1D**, *p* = 0.0001), as indicated by *post-hoc* analysis. No significant differences were detected in the time spent in the center of the open field (**Figure 1C**, *p* = 0.9718) and the total distance covered (**Figure 1D**, *p* = 0.8516) in the open field between control and insusceptible groups, as shown by *post-hoc* analysis.

### 3.2 Traumatic stress-induced transcriptional changes in PL and IL cortical regions

**Figure 2:**
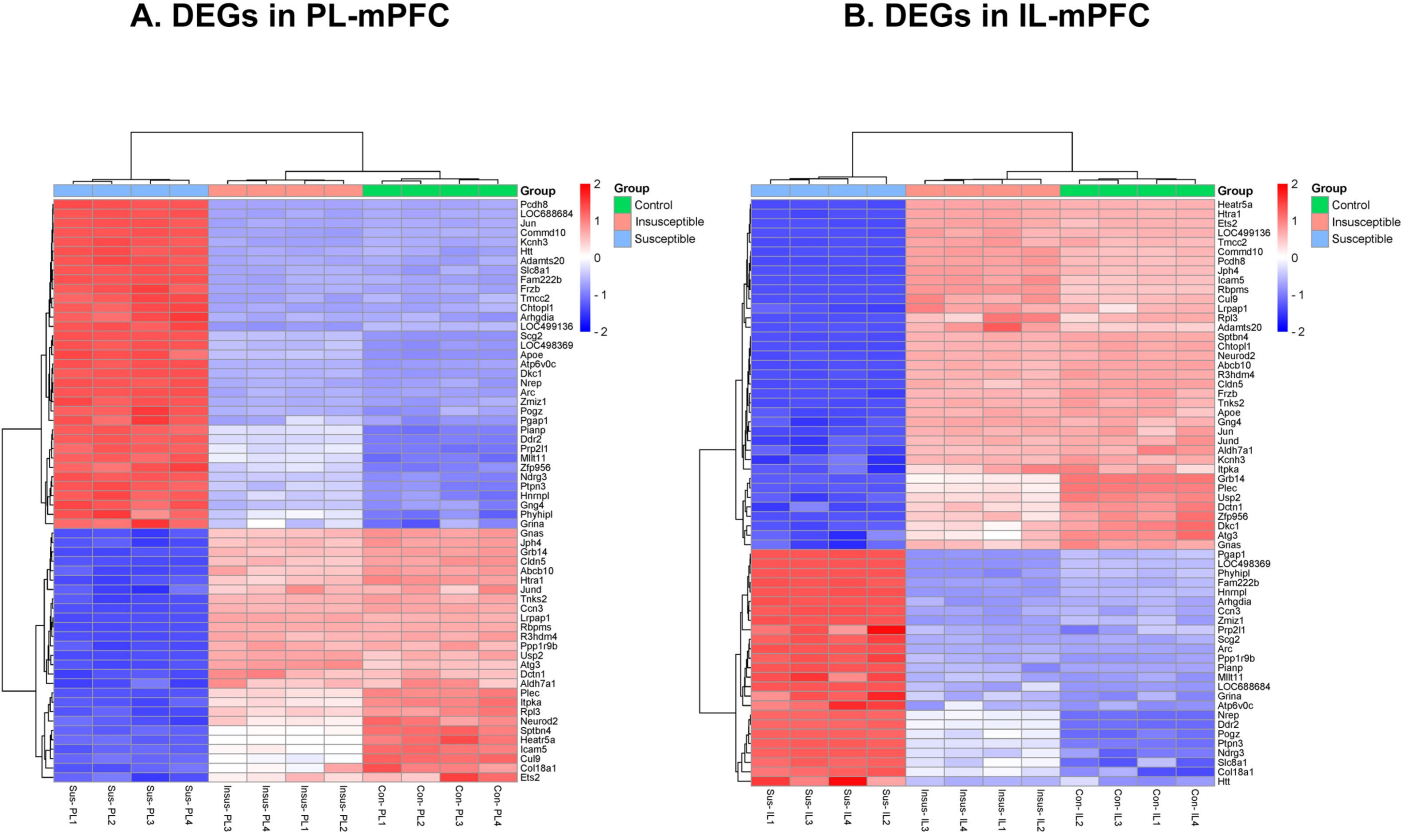
**(A, B)** Single prolonged stress (SPS) identified sixty-two differentially expressed genes (DEGs) significantly change in their expression between prelimbic (PL) and infralimbic (IL) regions of medial prefrontal cortex (mPFC) among the controls (con), susceptible (sus), and insusceptible (insus) groups. (n=4 in each group).

We performed RNA-seq to identify the transcriptional changes responsible for anxiety-like behavior in susceptible animals exposed to traumatic stress. A total of 2,478 DEGs (P < 0.05) were detected in PL and IL regions among the three groups (controls, susceptible, and insusceptible groups) (**Figure 1E**; **Supplementary Tables 1**, **2**). The majority of these DEGs were downregulated in PL and IL following SPS exposure. We observed a higher number of DEGs in the PL compared to the IL, with a predominantly downregulated expression. A larger number of overlapping DEGs between PL and IL were present in the susceptible group relative to the control and insusceptible groups, as illustrated by the Venn diagram (**Figure 1F**). Among the identified DEGs, only sixty-two DEGs (FDR adjusted p-value < 0.05) were significantly change in their expression in PL and IL regions among the controls, susceptible, and insusceptible groups (**Figure 2**). The identified DEGs in the PL and IL regions play crucial functional roles in various biological processes and are associated with anxiety and fear.

### 3.3 Traumatic stress affected KEGGs pathways leading to stress susceptibility and resilient phenotypes

**Figure 3:**
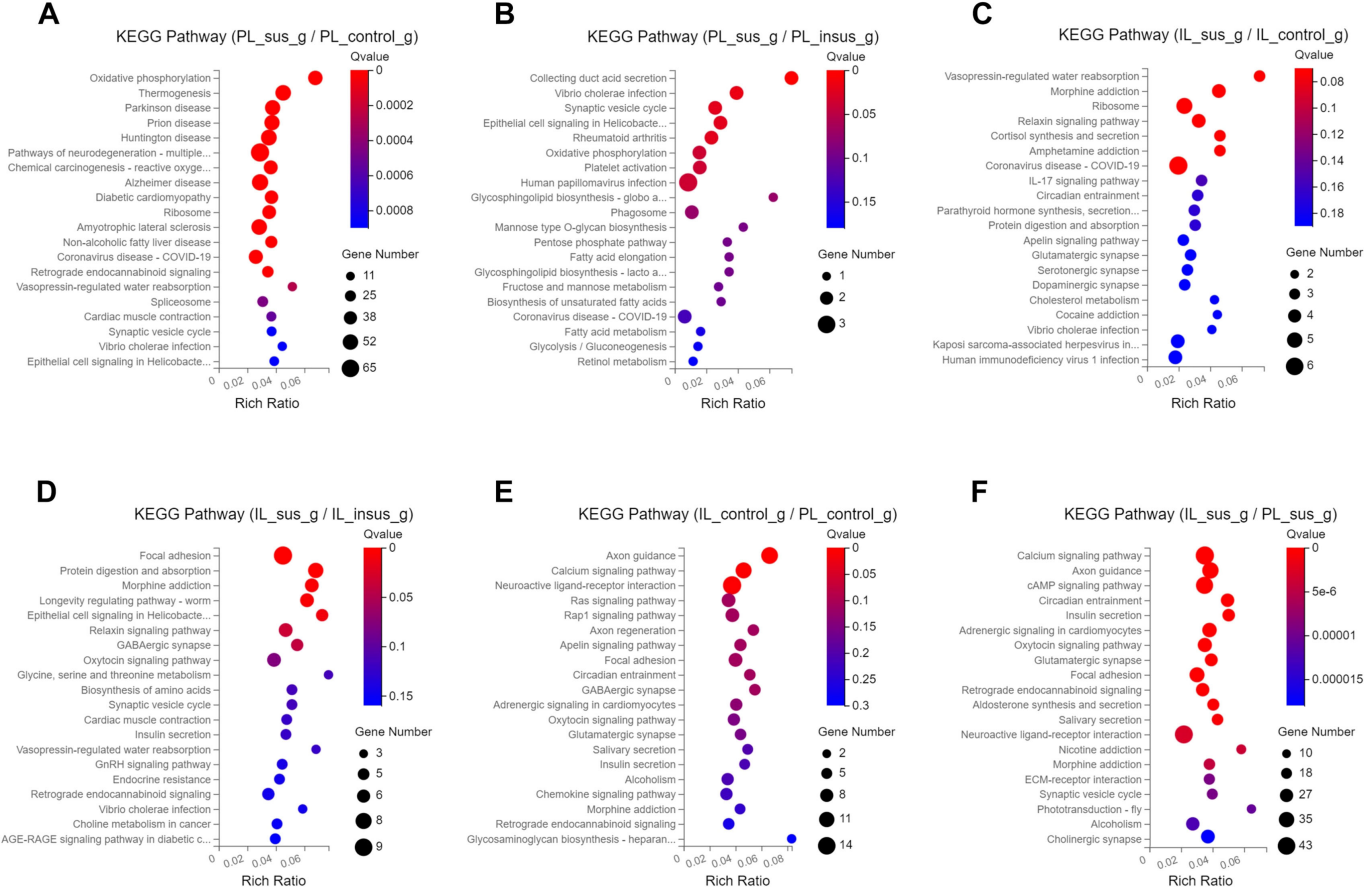
Kyoto Encyclopedia of Genes and Genomes (KEGG) pathways impacted by single prolonged stress (SPS). **(A)** DEGs in the PL-susceptible group, compared to the PL-control group, reveal an enrichment of KEGG pathways related to Parkinson’s, Huntington’s, and Alzheimer’s diseases, as well as pathways involved in neurodegeneration. **(B)** DEGs in the PL-susceptible group show less KEGG pathway enrichment when compared to the PL-insusceptible group. **(C, D)** In contrast to the IL-control and IL-insusceptible groups, the IL-susceptible group presents significant KEGG pathway enrichment associated with addiction-like behaviors, such as morphine and amphetamine addiction. Focal adhesion and relaxin signaling pathways are also more enriched in DEGs. **(E)** DEGs of PL and IL-control groups are involved in axon guidance, calcium signaling pathways, and neuroactive ligand-receptor interactions. **(F)** KEGG pathways affected by SPS in both PL-susceptible and IL-susceptible groups. Note: The X-axis represents the enrichment ratio (calculated as Rich Ratio = Term Candidate Gene Num/Term Gene Num), while the Y-axis denotes the KEGG pathway. Bubble size indicates the number of genes annotated to the KEGG pathway, and color represents enriched significance. The redder the color, the smaller the significance value (n=4 in each group).

To investigate the impact of SPS on KEGG pathways, we conducted KEGG analyses on the identified DEGs in the PL and IL mPFC. Our results indicated that SPS had differential effects on KEGG pathways in susceptible and insusceptible animals, which could contribute to the development of stress susceptibility and stress resilience phenotypes (**Figure 3**, **Supplementary Figure 1**). The DEGs in the PL-susceptible group, when compared to the PL-control group, exhibited enriched KEGG pathways related to Parkinson’s disease, Huntington’s disease, Alzheimer’s disease, and neurodegeneration processes (**Figure 3A**). The PL-susceptible group displayed fewer enriched KEGG pathways than the PL-insusceptible group (**Figure 3B**). The DEGs of the PL-control and PL-insusceptible groups did not differ in their regulation of KEGG pathways in the PL cortex. In the IL cortex, we observed that the IL-susceptible group, compared to the IL-control and IL-insusceptible groups, displayed significant enrichment of KEGG pathways involved in the regulation of relaxin and cortisol signaling pathways and addiction-like behaviors such as morphine and amphetamine addiction (**Figures 3C, D**).

We also compared KEGG pathways between the PL and IL cortices to identify underlying differences that could result in distinct functions of these regions in response to traumatic stress. We found that DEGs in both PL- and IL-control groups were enriched and upregulated in axon guidance, calcium signaling pathways, and neuroactive ligand-receptor interactions (**Figure 3E**). A comparative analysis of DEGs between PL and IL in susceptible groups revealed enrichment for axon guidance, calcium signaling pathways, and cAMP signaling pathways, which were upregulated in susceptible groups when comparing PL and IL cortices (**Figure 3F**). Addiction-related pathways were downregulated and displayed less DEG enrichment in controls when comparing PL and IL cortices. However, DEGs regulating the same addiction-related pathways were upregulated and exhibited increased enrichment for DEGs contributing to stress susceptibility in susceptible groups (**Figures 3E, F**).

### 3.4 Biological, cellular, and molecular functions affected by traumatic stress in the PL and IL cortices

**Figure 4:**
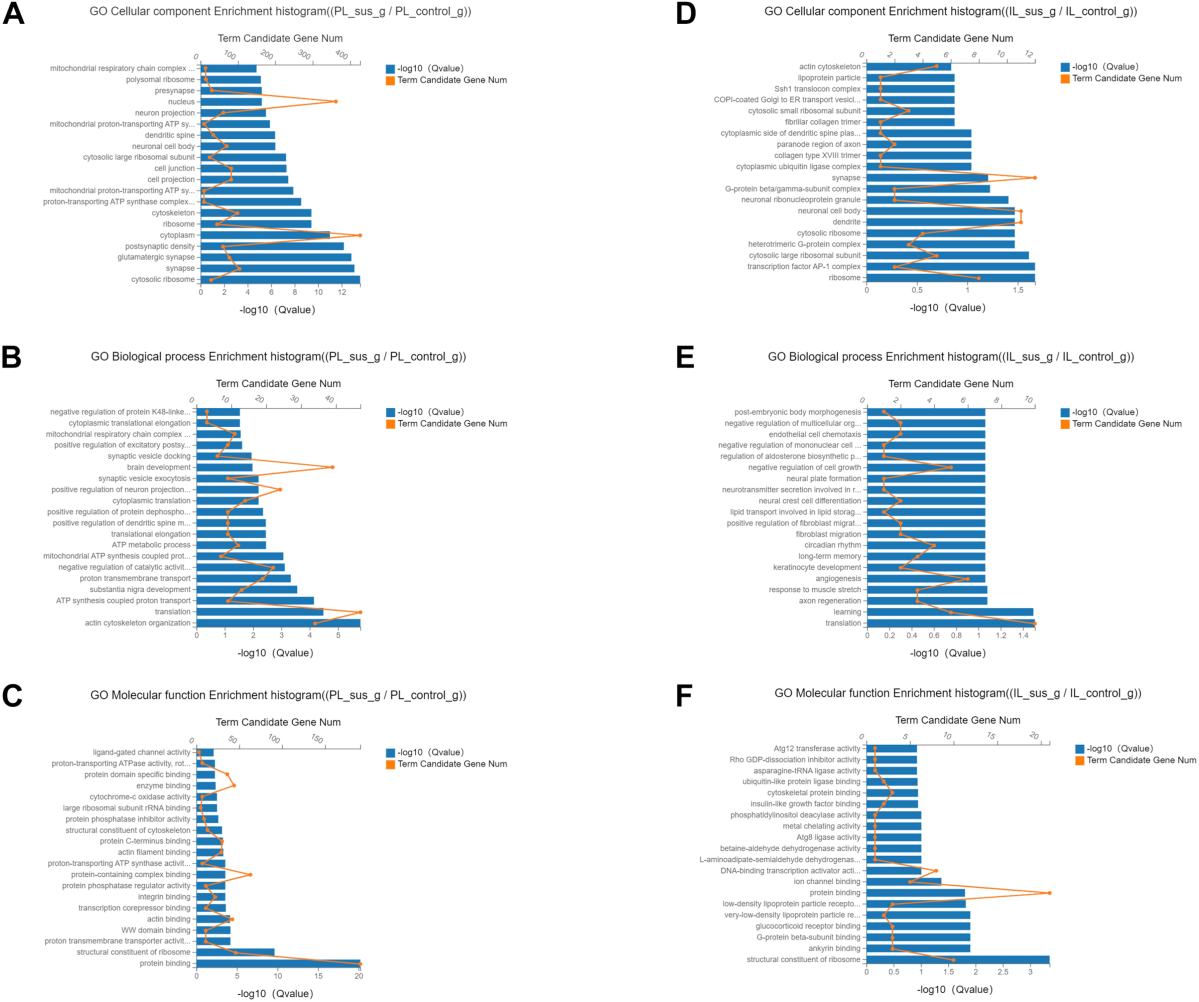
Classification of cellular, biological, and molecular functions based on Gene Ontology (GO) terms regulated by all DEGs. **(A–C)** Most identified DEGs showed GO enrichment for postsynaptic density protein, glutamatergic synapse, cytosolic ribosome, synapse formation, transmembrane transport protein, actin cytoskeleton reorganization, and protein binding in the PL-control versus PL-susceptible group. **(D–F)** In comparison to the IL-control group, the IL-susceptible group demonstrated enrichment of GO terms involved in synapse formation, neuronal activity, dendrite formation, axon regeneration, learning processes, and glucocorticoid receptor binding. (n=4 in each group) (FDR corrected *q*-value ≤ 0.05).

We performed GO analysis on the identified DEGs to ascertain the cellular, biological, and molecular functions regulated by all DEGs in the PL and IL cortices. The PL-control and insusceptible groups, compared to the PL-susceptible group, demonstrated GO enrichment for functions such as postsynaptic density protein, glutamatergic synapse, cytosolic ribosome, synapse formation, transmembrane transport protein, actin cytoskeleton reorganization, and protein binding (**Figures 4A–C**). In comparison to the IL-control and insusceptible groups, the IL-susceptible group displayed enrichment of GO terms associated with synapse formation, neuronal activity, dendrite formation, axon regeneration, learning processes, and glucocorticoid receptor binding (**Figures 4D–F**). A comparative analysis of GO enrichment revealed that the PL group regulated different subsets of GO terms compared to the IL group, and that DEG enrichment was more abundant in the PL region than in the IL region (**Figure 4**, **Supplementary Figure 2**).

## 4 Discussion

Numerous human and animal studies have consistently demonstrated the vital role of the PFC in decision-making and executive function (40, 41). The mPFC has been implicated in emotion, cognitive function, decision-making, and psychiatric disorders (42). Alterations in the function of two mPFC subregions (PL, and IL) have been observed in PTSD (12, 13). Given the involvement of the PL and IL regions in fear conditioning and extinction processes (43), as well as in stress-induced anxiety disorders, we conducted this study to elucidate the critical role of these two regions in traumatic stress. Utilizing an animal model of SPS, we observed the emergence of susceptible and insusceptible phenotypes. The susceptible animals exhibited anxiety-like behavior, as assessed by the EPM and OFT behavioral paradigms. Our RNA-seq data revealed numerous transcriptional changes in the PL region compared to the IL region. We found that SPS influenced DEGs-regulated KEGG and GO pathways in PL, which contributed to neurological disorders. Conversely, SPS affected a distinct set of KEGG and GO pathways in IL compared to PL, predisposing the animals to addiction-like behavior following traumatic stress. The insusceptible animals displayed transcriptional changes and pathways in PL and IL similar to controls. In conclusion, these findings suggest that SPS activates distinct transcriptional and molecular pathways in PL and IL, enabling differential coping mechanisms in response to the effects of traumatic stress. These molecular pathways underlie the stress-induced susceptible and insusceptible phenotypes observed in rats.

Previous preclinical and clinical investigations have reported transcriptional reorganization in the PFC and amygdala associated with depression and fear conditioning following traumatic stress (22, 43–45). In accordance with these studies, we also identified numerous transcriptomic reorganizations within the mPFC. The majority of these transcriptional changes were downregulated in the PL mPFC relative to the IL mPFC after SPS exposure. A recent study identified DEGs associated with cytokines, myotrophin, and glucocorticoid receptors in the locus coeruleus and nucleus accumbens (21). We detected DEGs in the PL and IL mPFC involved in regulating anxiety-like behavior in male rats; most of them were downregulated in the PL and IL mPFC of the susceptible groups compared to their control and insusceptible counterparts. These DEGs included Nr4a1, Rgs4, Rbfox1, Pcsk2, Pde1a, Cobl, Tmem132d, Bdnf, Lsm11, Junb, Htr2a, Slc39a10, and Trib1. We propose that the downregulation of the majority of these DEGs may underlie the anxiety-like behavior exhibited by male rats. Previous research has also implicated these DEGs in anxiety and depression disorders (20, 46–50).

SPS regulated the expression of 48 DEGs that modulate KEGG pathways (Ras signaling, calcium signaling, endocannabinoid signaling, oxidative phosphorylation signaling, and cAMP signaling) in the PL among control, susceptible, and insusceptible groups. The dysregulation of these KEGG pathways in the PL may increase susceptibility to neurodegenerative diseases, such as Alzheimer’s disease, Parkinson’s disease, and Huntington’s disease. Significant DEGs modulating these KEGG pathways in the PL of the mPFC) included UBB, Rps27, NADHB8, Tubb4a, Plcg1, Uqcrb, Rtn3, Gria1, and Htt genes. UBB (ubiquitin B) and Rps27 (ribosomal protein S27a) proteins were found to be significantly decreased in the hippocampus and cerebral cortex following traumatic brain injury (51). The significant downregulation of these DEGs after SPS in the PL suggests an increased susceptibility to traumatic brain injury. NDUF-B8 (ubiquinone oxidoreductase subunit B8) was significantly reduced in dopaminergic neurons, causing respiratory chain dysfunction in Parkinson’s disease patients (52). The Tubb4a (β-tubulin 4A) gene is involved in the myelination process in the brain, and mutations of Tubb4a have been associated with profound hypomyelination in human white matter disease (53). SPS reduced Tubb4a expression in the PL, potentially increasing the hypomyelination process and leading to a progressive loss of myelin in the brain.

Plcg1 (phospholipase C, gamma 1) is involved in the regulation of anxiety and depressive-like behavior in animals. Deficiency of Plcg1 in the forebrain resulted in hyperactivity, decreased anxiety, and depressive-like behavior (54). Our study found that SPS increased Plcg1 expression in PL-susceptible animals, which could heighten anxiety and depressive-like behavior following traumatic stress. The upregulation of Uqcrb (ubiquinol-cytochrome c reductase binding protein) has been shown to provide protection against stress and depression (55). However, SPS decreased Uqcrb expression in the PL of susceptible rats compared to controls and insusceptible rats, indicating an increased susceptibility of the PL to stress and depression. Rtn3 (reticulon 3) is implicated in traumatic brain injury, and its overexpression promotes neurite outgrowth in the brain (56). Our findings revealed downregulation of Rtn3 in the PL-susceptible group, which may contribute to traumatic brain injury following stress. SPS increased Gria1 (glutamate ionotropic receptor AMPA type subunit 1) expression in the PL of susceptible rats compared to control and insusceptible rats. Prior studies have reported elevated Gria1 expression in schizophrenia and neurodevelopmental disorders (57, 58). SPS reduced Htt (huntingtin) gene expression, which has been linked to cerebral ischemia (59), Huntington’s disease (60), and Parkinson’s disease (61).

In comparison to the PL region, we identified 28 DEGs in the IL region involved in specific KEGG pathways, which led to dysfunction in relaxin and GABAergic signaling as well as susceptibility to drug addiction (morphine addiction, amphetamine addiction) in the susceptible group relative to the control and insusceptible groups. Notably, the DEGs with a significant difference in the relaxin signaling pathway included Jun, Col4a2, GNAS, and Mapk8. Jun, an AP-1 transcription factor subunit, is implicated in the relaxin signaling pathway, and its expression was elevated in the IL-susceptible group following SPS exposure when compared to the control and insusceptible groups. Jun plays a crucial role in enhancing the transcriptional response of Schwann cells after nerve injury (62). Additionally, SPS increased Col4a2 (collagen type IV alpha 2 chain) gene expression in IL, with mutations in Col4a2 being associated with hemorrhagic stroke (63). SPS downregulated Mapk8 (mitogen-activated protein kinase 8) expression in the IL-susceptible group relative to the control and insusceptible groups. Mapk8 is involved in neuronal development via the JNK pathway (64), and its downregulated expression by SPS could impact the developmental process in neurons. Moreover, elevated expression of Gnas (G-protein alpha subunit) is linked to increased susceptibility to anxiety disorders in humans (47). SPS augmented Gnas expression in IL-susceptible rats, leading to anxiety-like behavior after traumatic stress.

Furthermore, SPS reduced the expression of DEGs (Gabarapl1, Gabrb2, Gabra3, Gnb1, and Gng3) involved in GABAergic signaling in the IL-susceptible group. This decreased expression of DEGs could affect GABAergic synaptic transmission, GABA receptor binding activity, and brain development (65, 66). In the IL region, Jun, Arc, and Gnas DEGs were enriched in KEGG pathways related to amphetamine addiction. The downregulation of these DEGs in IL by SPS could influence synaptic plasticity, glutamatergic synapses, and the functional connectivity of corticolimbic structures, ultimately resulting in amphetamine addiction (67–69). Lastly, the DEGs with significantly higher expression in IL following SPS were Pde8b and Gnas. These DEGs could impact motor function in the CNS and the reconsolidation of morphine reward memories (70, 71).

It is well documented that a key mechanism contributing to the fast-acting effects of antidepressants is the enhancement of neuroplasticity (72–74). In line with the current findings, particularly those related to the stress-susceptible group, DEGs showed reduced enrichment in synapse-related functions, such as PSD proteins, transmembrane transport proteins, and actin reorganization. The serotonin and norepinephrine reuptake inhibitors (SNRIs) play a significant role as a fast-acting antidepressants (75). The identified DEGs in PL and IL susceptible groups could activate the molecular signaling cascades that decreased excitatory neurotransmission via NMDAR (76), AMPAR (77), and mTORC1 (78) signaling.

In conclusion, the ten-day SPS procedure produced anxiety-like behavior in rats, differentiated into resilient and susceptible phenotypes. We found divergent responses among stressed rats subjected to the SPS procedure. RNA-seq analysis identified sixty-two significant DEGs playing differential role in PL and IL mPFC, contributing to the resilient and susceptible phenotypes. The DEGs in the PL and IL susceptible groups exhibited reduced enrichment for cellular, biological, and molecular functions based on GO pathways, which may underlie the increased anxiety-like behavior observed in susceptible rats.

## 5 Limitations of the study

The results of this study are limited to the groups analyzed in this manuscript. For RNA-Seq, we only used the first four animals from each group based on their AI in control, insusceptible, and susceptible groups. We have performed this study exclusively on male rats to completely understand the effect of SPS on mPFC transcriptome. We excluded female rats from this study due to the fact that the estrogen fluctuation during the estrous cycle might interfere with the effect of SPS on behavior and transcriptomic profile of mPFC.

## Data Availability

The RNA-Seq data in this study are deposited in the NCBI SRA repository with accession number PRJNA1001887 and are accessible at the following link: https://www.ncbi.nlm.nih.gov/sra/PRJNA1001887.

## Supplementary material

Supplementary Figure 1 KEGG pathways regulated by DEGs of PL and IL in control, susceptible, and insusceptible groups. **(A–C)** Most DEGs were involved in organismal systems and human diseases, while DEGs involved in cellular processes, genetic and environmental processes decreased in the PL-susceptible group compared to PL-control and insusceptible groups. Metabolism-regulating DEGs show a different pattern in the PL-control group *vs*. PL-susceptible group and PL-susceptible group *vs*. PL-insusceptible group. **(D–F)** KEGG pathways were enriched in organismal processes, metabolism, and human diseases in both groups (IL-susceptible compared with IL-control and IL-insusceptible groups). DEGs involved in cellular, genetic, and environmental processes in the IL-susceptible group showed different responses to SPS compared to IL-control and insusceptible groups.

Supplementary Figure 2 GO classification of DEGs based on comparative analysis of cellular, biological, and molecular functions between PL and IL in control, susceptible, and insusceptible groups.
