# Supplementary material for: Single prolonged stress induces behavior and transcriptomic changes in the medial prefrontal cortex to increase susceptibility to anxiety-like behavior in rats
Source: Front Psychiatry. 2024 Nov 19;15:1472194. doi: 10.3389/fpsyt.2024.1472194 (PMC11611810; doi:10.3389/fpsyt.2024.1472194)
Supplement: Supplementary file 1 [file Table1.docx]

| **Supplementary Table 1:** The overlapping KEGGS pathways significantly enriched in control, insusceptible and susceptible groups in PL-mPFC | | | | | | |
| --- | --- | --- | --- | --- | --- | --- |
| **KEGG Pathways** | **Term Candidate Gene Num** | **Total Candidate Gene Num** | **Total Gene Num** | **Rich Ratio** | **P value** | **Q value** |
| Parkinson disease | 47 | 257 | 8835 | 0.183 | 3.11E-14 | 3.23E-12 |
| Prion disease | 47 | 260 | 8835 | 0.181 | 4.90E-14 | 3.81E-12 |
| Huntington disease | 50 | 294 | 8835 | 0.170 | 8.73E-14 | 5.43E-12 |
| Pathways of neurodegeneration - multiple diseases | 65 | 464 | 8835 | 0.140 | 1.86E-13 | 9.62E-12 |
| Chemical carcinogenesis - reactive oxygen species | 39 | 221 | 8835 | 0.176 | 1.71E-11 | 7.60E-10 |
| Alzheimer disease | 53 | 378 | 8835 | 0.140 | 3.85E-11 | 1.50E-09 |
| Diabetic cardiomyopathy | 36 | 201 | 8835 | 0.179 | 6.88E-11 | 2.38E-09 |
| Ribosome | 38 | 222 | 8835 | 0.171 | 8.08E-11 | 2.51E-09 |
| Amyotrophic lateral sclerosis | 49 | 357 | 8835 | 0.137 | 4.77E-10 | 1.35E-08 |
| Non-alcoholic fatty liver disease | 28 | 157 | 8835 | 0.178 | 1.06E-08 | 2.75E-07 |
| Coronavirus disease - COVID-19 | 40 | 318 | 8835 | 0.126 | 2.34E-07 | 5.61E-06 |
| Retrograde endocannabinoid signaling | 24 | 144 | 8835 | 0.167 | 4.50E-07 | 1.00E-05 |
| Vasopressin-regulated water reabsorption | 11 | 44 | 8835 | 0.250 | 1.25E-05 | 2.60E-04 |
| Morphine addiction | 13 | 92 | 8835 | 0.141 | 0.001 | 0.014 |
| cAMP signaling pathway | 23 | 217 | 8835 | 0.106 | 0.001 | 0.014 |
| Hepatocellular carcinoma | 20 | 178 | 8835 | 0.112 | 0.001 | 0.014 |
| Oxytocin signaling pathway | 18 | 155 | 8835 | 0.116 | 0.001 | 0.016 |
| Dopaminergic synapse | 16 | 132 | 8835 | 0.121 | 0.001 | 0.018 |
| Neurotrophin signaling pathway | 15 | 120 | 8835 | 0.125 | 0.001 | 0.018 |
| Collecting duct acid secretion | 6 | 27 | 8835 | 0.222 | 0.002 | 0.026 |
| Glutamatergic synapse | 14 | 114 | 8835 | 0.123 | 0.002 | 0.028 |
| mTOR signaling pathway | 17 | 157 | 8835 | 0.108 | 0.003 | 0.039 |
| Rap1 signaling pathway | 21 | 213 | 8835 | 0.099 | 0.004 | 0.041 |
| Adherens junction | 10 | 72 | 8835 | 0.139 | 0.004 | 0.041 |
| Focal adhesion | 20 | 200 | 8835 | 0.100 | 0.004 | 0.041 |
| GnRH signaling pathway | 11 | 90 | 8835 | 0.122 | 0.007 | 0.069 |
| GABAergic synapse | 11 | 91 | 8835 | 0.121 | 0.008 | 0.073 |
| EGFR tyrosine kinase inhibitor resistance | 10 | 80 | 8835 | 0.125 | 0.009 | 0.079 |
| Oocyte meiosis | 13 | 120 | 8835 | 0.108 | 0.010 | 0.089 |
| Apelin signaling pathway | 14 | 137 | 8835 | 0.102 | 0.013 | 0.109 |
| Thyroid hormone signaling pathway | 13 | 124 | 8835 | 0.105 | 0.013 | 0.109 |
| Long-term depression | 8 | 61 | 8835 | 0.131 | 0.014 | 0.111 |
| Salmonella infection | 13 | 92 | 8835 | 0.141 | 0.034 | 0.014 |
| Glioma | 23 | 217 | 8835 | 0.106 | 0.038 | 0.014 |
| Relaxin signaling pathway | 20 | 178 | 8835 | 0.112 | 0.004 | 0.014 |
