# Supplementary material for: Single prolonged stress induces behavior and transcriptomic changes in the medial prefrontal cortex to increase susceptibility to anxiety-like behavior in rats
Source: Front Psychiatry. 2024 Nov 19;15:1472194. doi: 10.3389/fpsyt.2024.1472194 (PMC11611810; doi:10.3389/fpsyt.2024.1472194)
Supplement: Supplementary file 2 [file Table2.docx]

| **Supplementary Table 2:** The overlapping KEGGS pathways significantly enriched in control, insusceptible and susceptible groups in IL-mPFC | | | | | | |
| --- | --- | --- | --- | --- | --- | --- |
| **KEGG Pathway Term Desc** | **Term Candidate Gene Num** | **Total Candidate Gene Num** | **Total Gene Num** | **Rich Ratio** | **P value** | **Q value** |
| Morphine addiction | 4 | 39 | 8835 | 0.043 | 0.001 | 0.076 |
| Vasopressin-regulated water reabsorption | 3 | 39 | 8835 | 0.068 | 0.001 | 0.076 |
| Relaxin signaling pathway | 4 | 39 | 8835 | 0.031 | 0.002 | 0.076 |
| Coronavirus disease - COVID-19 | 6 | 39 | 8835 | 0.019 | 0.002 | 0.076 |
| Ribosome | 5 | 39 | 8835 | 0.023 | 0.003 | 0.076 |
| Cortisol synthesis and secretion | 3 | 39 | 8835 | 0.044 | 0.003 | 0.076 |
| Amphetamine addiction | 3 | 39 | 8835 | 0.044 | 0.003 | 0.076 |
| IL-17 signaling pathway | 3 | 39 | 8835 | 0.033 | 0.007 | 0.151 |
| Circadian entrainment | 3 | 39 | 8835 | 0.031 | 0.009 | 0.162 |
| Protein digestion and absorption | 3 | 39 | 8835 | 0.029 | 0.010 | 0.162 |
| Parathyroid hormone synthesis, secretion and action | 3 | 39 | 8835 | 0.029 | 0.011 | 0.162 |
| Glutamatergic synapse | 3 | 39 | 8835 | 0.026 | 0.014 | 0.181 |
| Kaposi sarcoma-associated herpesvirus infection | 4 | 39 | 8835 | 0.019 | 0.015 | 0.181 |
| Serotonergic synapse | 3 | 39 | 8835 | 0.024 | 0.017 | 0.181 |
| Cocaine addiction | 2 | 39 | 8835 | 0.043 | 0.018 | 0.181 |
| Human immunodeficiency virus 1 infection | 4 | 39 | 8835 | 0.017 | 0.019 | 0.181 |
| Cholesterol metabolism | 2 | 39 | 8835 | 0.041 | 0.020 | 0.181 |
| Dopaminergic synapse | 3 | 39 | 8835 | 0.023 | 0.020 | 0.181 |
| Vibrio cholerae infection | 2 | 39 | 8835 | 0.039 | 0.021 | 0.181 |
| Apelin signaling pathway | 3 | 39 | 8835 | 0.022 | 0.022 | 0.181 |
| Mineral absorption | 2 | 39 | 8835 | 0.035 | 0.026 | 0.202 |
| Endocrine and other factor-regulated calcium reabsorption | 2 | 39 | 8835 | 0.034 | 0.028 | 0.205 |
| Cushing syndrome | 3 | 39 | 8835 | 0.019 | 0.032 | 0.227 |
| Wnt signaling pathway | 3 | 39 | 8835 | 0.018 | 0.036 | 0.241 |
| Epithelial cell signaling | 2 | 39 | 8835 | 0.029 | 0.037 | 0.241 |
| MAPK signaling pathway | 4 | 39 | 8835 | 0.014 | 0.039 | 0.246 |
| Alcoholism | 3 | 39 | 8835 | 0.017 | 0.043 | 0.258 |
