# Supplementary figures and images for: Single prolonged stress induces behavior and transcriptomic changes in the medial prefrontal cortex to increase susceptibility to anxiety-like behavior in rats
Source: Front Psychiatry. 2024 Nov 19;15:1472194. doi: 10.3389/fpsyt.2024.1472194 (PMC11611810; doi:10.3389/fpsyt.2024.1472194)

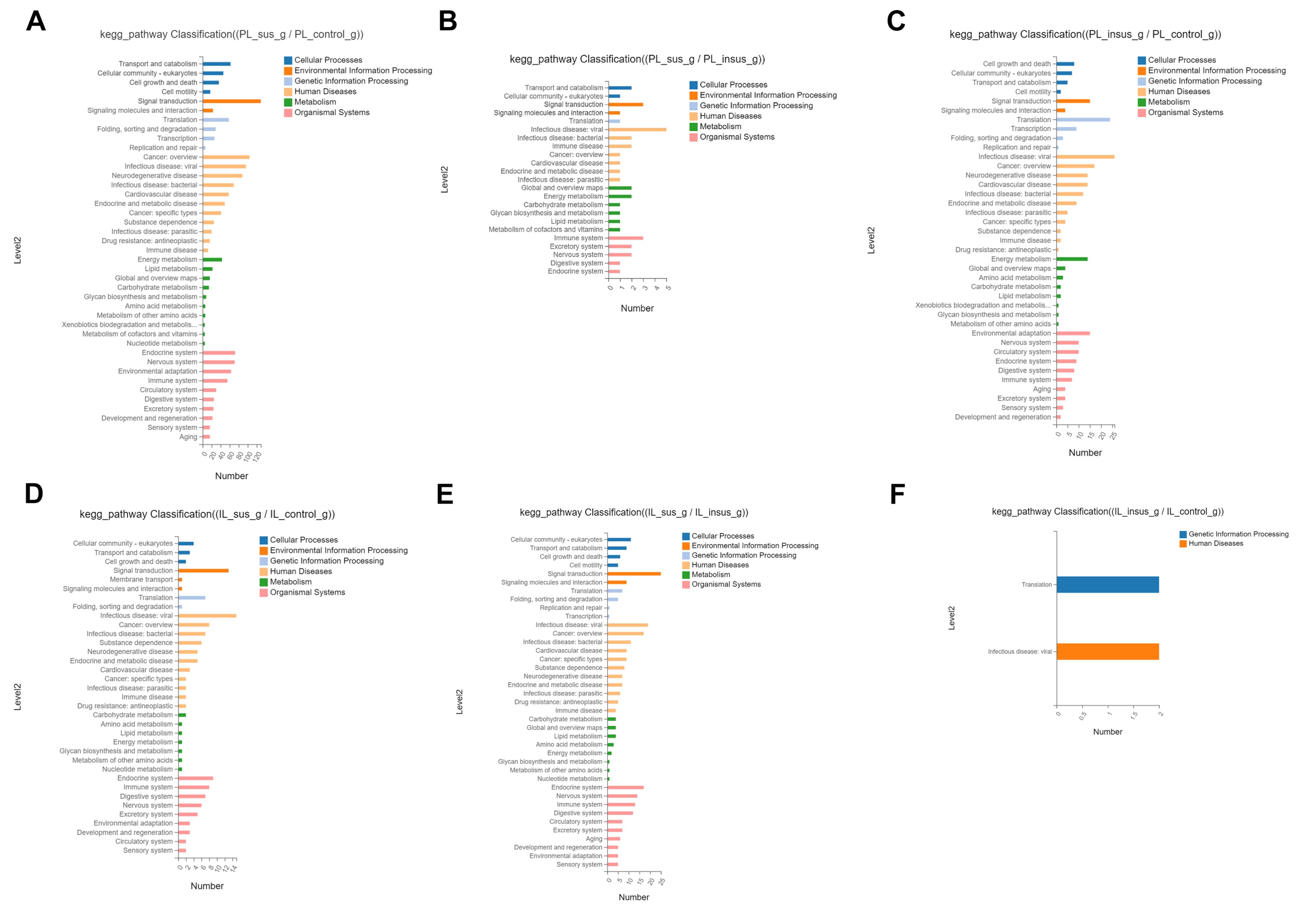

Supplement: Supplementary Figure 1 — KEGG pathways regulated by DEGs of PL and IL in control, susceptible, and insusceptible groups. (A–C) Most DEGs were involved in organismal systems and human diseases, while DEGs involved in cellular processes, genetic and environmental processes decreased in the PL-susceptible group compared to PL-control and insusceptible groups. Metabolism-regulating DEGs show a different pattern in the PL-control group vs. PL-susceptible group and PL-susceptible group vs. PL-insusceptible group. (D–F) KEGG pathways were enriched in organismal processes, metabolism, and human diseases in both groups (IL-susceptible compared with IL-control and IL-insusceptible groups). DEGs involved in cellular, genetic, and environmental processes in the IL-susceptible group showed different responses to SPS compared to IL-control and insusceptible groups. [file Image1.jpeg]

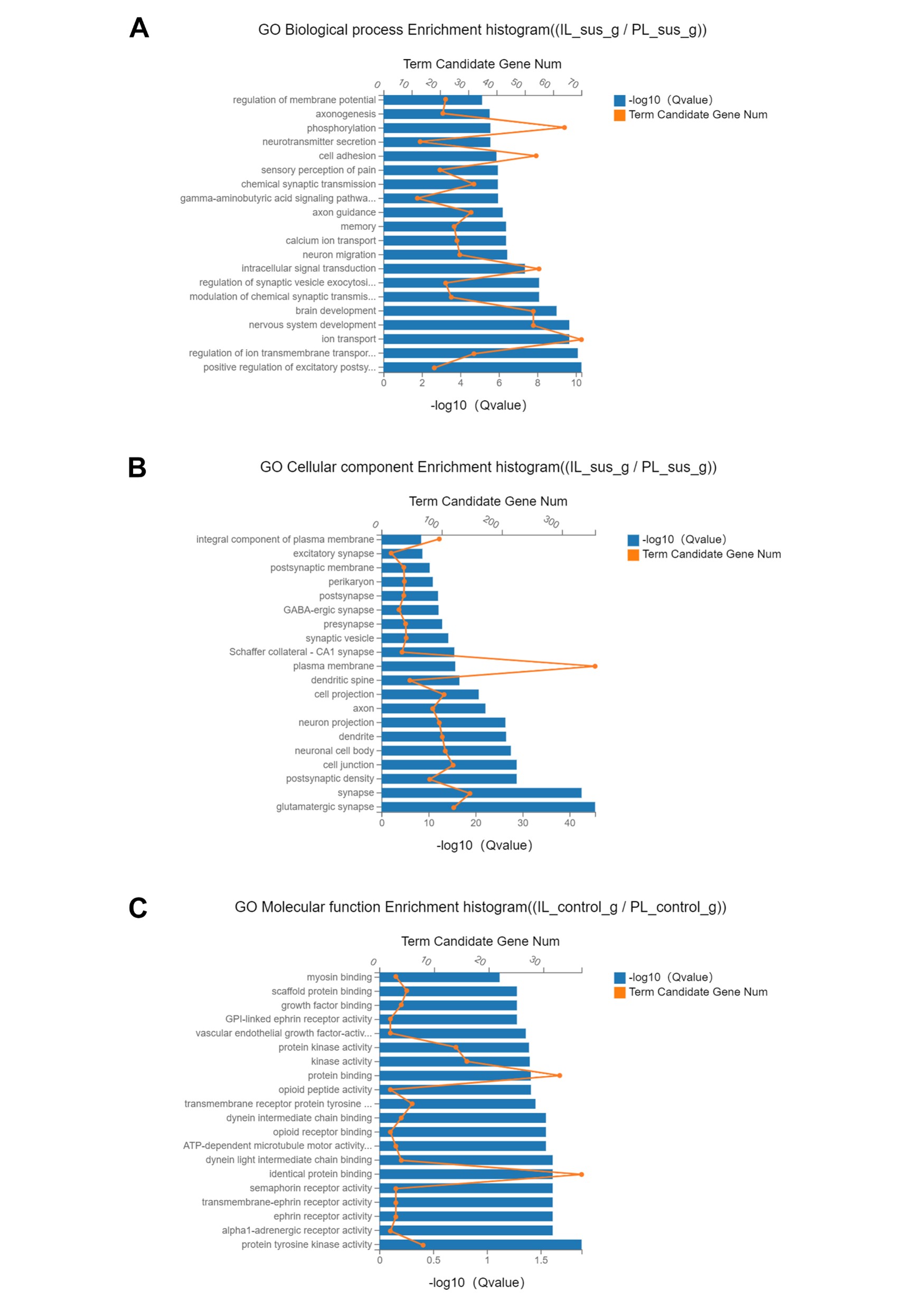

Supplement: Supplementary Figure 2 — GO classification of DEGs based on comparative analysis of cellular, biological, and molecular functions between PL and IL in control, susceptible, and insusceptible groups. [file Image2.jpeg]
